# EHMTI-0347. Migraine prevalence in patients aged up to 50 with acute cerebrovascular insult (CVI) treated in St.Sava Hospital during 2012

**DOI:** 10.1186/1129-2377-15-S1-L2

**Published:** 2014-09-18

**Authors:** N Milovanovic Kovacevic, N Zaric, M Savic

**Affiliations:** 1 Intensive Care, Hospital for Cerebrovascular Diseases Sveti Sava, Belgrade, Serbia

## Objective

of this study was to determine percentage incidence of migraine in patients whit acute CVI compared to the population of patients with acute CVI without pre-morbid migraine,all of whom were aged up to 50 and 50 .Migraine prevelance up to the above said age is the largest in extent,whereas the incidence of other cerebrovascular disease risk factors is the smallest.This is why other factors minimally affected the result set forth as the objective of this study.The goal in this study was to determine migraine prevalence in patients aged up to 50 with acute CVI,as well as to prove migraine infraction within this population.

## Methods

Statistical processing of the data obtained from the computerized database of St.Sava Hospital was applied.In the period from 1 January 2012 to 31 December 2012.

## Result

Hetero-anamnestic and auto-anamnestic data revealed that,within this age group of patients 45 female patients used to have migraine headaches.Out of them,3 patients suffered from migraine with aura ,while another woman aged 38 suffered from migraine with aura and had neurological deficit in terms of hemiparesis on the right within the aura.The neurological deficit was retained even after the migraine attack.Neuro-imaging methods confirmed the left temporal-parietal position of an ischemic lesion.This case represents the only confirmed instance of migraine infarction.

## Conclusion

This study showed that migraine prevalence in patients with acute CVI is not larger than prevalence in general population.In addition,a single instance of migraine infraction was confirmed in a female patient in her 30s who suffered from migraine with aura.

No conflict of interest.

